# Development and evaluation of an integrated diabetes-periodontitis nutrition and health education module

**DOI:** 10.1186/s12909-021-02721-9

**Published:** 2021-05-17

**Authors:** Nor Aini Jamil, Shin Hwa Chau, Nabihah Iman Abdul Razak, Iffa Izzwani Shamsul Kamar, Shahida Mohd-Said, Haslina Rani, Mohd Jamil Sameeha

**Affiliations:** 1 Centre for Community Health Studies (ReaCH), Faculty of Health Sciences, Universiti Kebangsaan Malaysia, 50300 Kuala Lumpur, Malaysia; 2 Department of Restorative Dentistry, Faculty of Dentistry, Universiti Kebangsaan, Kuala Lumpur, 50300 Malaysia; 3 Department of Family Oral Health, Faculty of Dentistry, Universiti Kebangsaan, Kuala Lumpur, 50300 Malaysia

**Keywords:** Diabetes wellness, Health education, Oral health, PEMAT, Actionability

## Abstract

**Background:**

A good understanding of the bi-directional relationship between diabetes and periodontitis is essential to ensure the successful management of both diseases. This study aimed to develop and evaluate an integrated diabetes-periodontitis nutrition and health education module.

**Methods:**

The module was developed as an iterative and review process by five experts in nutrition and dietetics, periodontics, and dental public health. It consisted of three phases: (i) needs assessment on module contents and characteristics, (ii) module development and (iii) module evaluation by experts. Twelve healthcare professionals aged between 30 and 53 years (average 13.5 years of working experience) validated the module contents and its comprehensibility using the Patient Education Materials Assessment Tool for printable materials (PEMAT-P) and audio-visual materials (PEMAT-A/V). Scores of 0 (disagree) or 1 (agree) were given for sets of understandability and actionability statements and presented as a total percentage.

**Results:**

Seventeen infographic-flip charts and 13 short-videos were developed in the Malay language and grouped into four topics: (i) Introduction to Diabetes and Periodontitis, (ii) Diabetes and Periodontitis Care, (iii) Lifestyle Modification, and (iv) Myths and Facts. Flip charts were rated between 76-100% for understandability and 80–100% for actionability, while videos rated between 90-100% for understandability and 100% for actionability, respectively.

**Conclusion:**

Overall, the newly developed module ranked high median scores for understandability and actionability. This finding reflects positive acceptance of the integrated module among the various healthcare professionals involved in managing patients with diabetes and periodontitis.

## Background

Type 2 diabetes mellitus (T2DM) is the most common form of diabetes among adults. Approximately 463 million people globally had diabetes in 2019, and this number is projected to rise to 700 million by 2045 [1]. At the same time, over 10.8% of the world’s population is affected by severe periodontitis [2], which is the sixth complication of T2DM [3]. Periodontitis occurs in the tooth-supporting structures known as periodontium due to untreated gingivitis, i.e., inflammation that begins in the gums due to irritation caused by toxins produced by dental plaque [4–6]. In Malaysia, 18.3% of adults have diabetes and 23.6% pre-diabetes [7]. Meanwhile, periodontitis affects nearly half of Malaysians aged 16 years and older, with 30.3% having moderate and 18.2% severe periodontitis [8]. Periodontitis management imposes a significant economic burden on the country [9].

For a long time, healthcare professionals have emphasised managing the cause of T2DM through improvements of unhealthy lifestyles and management of obesity [10–12]. More recently, there is increasing highlight on the impact of T2DM and periodontitis bilateral relationship on disease progression and treatment outcomes [13–16]. Since then, management of both diseases has focused on integrating diabetic care in health clinics alongside periodontitis therapy in dental clinics.

The key to ensuring the success of this integrated management of diabetes in patients with and at risk of periodontitis depends highly on the awareness of patients and the healthcare professionals and treatment planning. However, the awareness of the diseases’ relationship among the population and non-dental healthcare professionals is still low [17–20]. Patients with diabetes and non-dental healthcare professionals perceived dental care as less critical [21]. The current team approach of diabetes wellness modules does not emphasise oral health care [22]. This paper reports the collaborative effort in developing and evaluating a new nutrition and health education module designed for healthcare professionals use during consultation sessions with diabetes and periodontitis patients.

## Methods

### Study design

This study consisted of three phases: 1) needs assessment on module contents and characteristics, 2) development of nutrition and health education module and 3) evaluation of the module. Ethics approval was obtained from the University’s Research Ethics Committee (reference: UKM PPI/111/8/JEP-2020-106), and all participants gave written informed consent before the start of the study.

### Phase 1: needs assessment on module contents and characteristics

An extensive literature review was initially conducted to determine the potential contents of interest for module development purposes. A literature search via electronic databases including EBSCOhost and Google was carried out to find relevant T2DM and/or periodontitis guidelines, articles and educational materials between August 2019 to January 2020. Information was extracted and summarised in a table. Subsequently, semi-structured in-depth interviews were conducted among healthcare professionals from a public university hospital in Kuala Lumpur to seek opinions on the module’s contents and design. The interviews were carried out in English and Malay languages by a trained interviewer (C.S.H.) either in private office rooms, diet counselling room or dental clinics. A digital voice recorder (Sony ICD-PX470, Sony Corporation, Japan) and an Android Huawei mobile phone (Huawei Y9, Huawei Device Co., Ltd., China) were used to record all interviews, in addition to written field notes during the interview sessions, which were later summarised.

### Phase 2: module development

The module development involved an iterative design and review process by five experts comprising of two dietitians (N.A.J and C.S.H), a nutritionist (M.J.S), a periodontist (S.M-S), and a dental public health specialist (H.R). The module was written in the Malay language and consisted of 17 units under four topics: (i) Introduction to Diabetes and Periodontitis, (ii) Diabetes and Periodontitis Care, (iii) Lifestyle Modifications and (iv) Myths and Facts. Based on the phase 1 recommendations, two modes of delivery were chosen: (i) printed material via flip chart for healthcare professionals’ use during consultation sessions with patients with diabetes and periodontitis disease, and (ii) audio-visual aids via short videos as educational materials for patients’ references. All flip charts were developed using Canva graphic design platform (Canva Pty. Ltd), whereas videos were made by Powtoon, The Visual Communication Platform (Powtoon Ltd.). The module used simple terms and language, attractive and colourful illustrations, pictures with captions, and culturally suitable examples. The illustrations and pictures used in the module were obtained from the Freepik graphic design website (Freepik Company S.L.).

### Phase 3: module evaluation

A panel of interdisciplinary healthcare experts consisting of citizens with a minimum of 3 years of experience in diabetes care and/or periodontitis from medical universities and hospitals in Kuala Lumpur was invited to evaluate the module. All participants involved in the previous phase 1 were contacted through emails to participate in this study. An additional six new healthcare professionals (2 dietitians and 4 dental specialists) were contacted to participate in the evaluation phase. The number of experts evaluating the module in this study is more than the recommended numbers suggested by previous studies of at least five expert panels [23, 24].

The evaluation process was performed during the Coronavirus disease pandemic (COVID-19) and Restriction Movement Order in Malaysia (February 2020 until April 2020), where all communications were done online via emails, phone and video calls. Participants were provided with a link to access a Google Drive folder that contained: (i) an Introduction File that briefly introduced the module and provided instructions on how to conduct the evaluation process, and (ii) two other folders containing flip charts in pdf form and videos labelled according to module units.

For each material, an evaluation form was provided for an independent review process. The Malay version of the Patient Education Materials Assessment Tool for printable materials (PEMAT-P), which comprises 17 statements for understandability and 7 for actionability, was used to evaluate each flip chart [25]. ‘Understandability’ is defined as the ability of people from diverse backgrounds with varying health literacy abilities to comprehend educational materials and extract key messages, while ‘actionability’ is the respondents’ ability to identify what can take actions based on educational material information [26]. Subsequently, a questionnaire for audio-visual materials (PEMAT-A/V) consisted of 13 statements for understandability and 4 for actionability was used to evaluate each video. Each item on PEMAT scored as either 1 (agree), 0 (disagree), or N/A (not applicable to the material). The total understandability and actionability scores were calculated and presented in percentage (%). A higher score of the material’s understandability and actionability indicates that the educational material is easy to understand and action.

## Results

### Phase 1: needs assessment on module contents and characteristics

From the literature search, fifteen topics were proposed for the integrated module for healthcare professionals’ use during consultation sessions with patients with diabetes and periodontitis disease (Table 1). Then, the table was presented during interview sessions with fifteen healthcare professionals (mean age 41.9 years, SD 8.0) consisted of dietitians, diabetes educators, dental professionals (dental specialist and dental officer), an endocrinologist, a health psychologist, and a pharmacist (Table 2). Majority of them were female (87%), with an average service duration of 15 years (SD 6.6) in the current institution.

**Table 1:** Proposed topics and contents for the integrated diabetes-periodontitis nutrition and health education module

No.	Topics	Contents
1.	Introduction to Type 2 Diabetes Mellitus (T2DM)	● Definition of diabetes ● Causes of diabetes ● Sign and symptoms of diabetes ● Risk factors of diabetes ● Complications of diabetes ● Risk of pre-diabetes
2.	Introduction to Periodontitis	● Definition of periodontitis ● Causes of periodontitis ● Signs and symptoms of periodontitis ● Risk factors of periodontitis ● Complications of periodontitis
3.	Diabetes and Periodontitis	● Bi-directional relationship between diabetes and periodontitis ● Hyperglycaemia and oral health ● Diabetes, periodontitis and mouth breath
4.	Treatment	● Treatment for diabetes ● Treatment for dental disease
5.	Blood Sugar Monitoring	● Target of blood sugar profile ● Importance of good glycaemic control
6.	Weight Management	● Principles of weight management ● Target for weight reduction ● Readiness to change
7.	Dietary Management	● Healthy eating ● Carbohydrates and carbohydrate exchanges ● Added sugar ● Dietary fibre ● Antioxidants ● Menu planning ● Food label ● Tips for healthy cooking ● Tips for eating outside
8.	Physical Activity & Exercise	● Benefits of exercise ● Types of exercise ● Duration of exercise ● How to achieve 10,000 steps ● Simple tips to be physically active ● Precautions to hypoglycaemia during exercise
9.	Ways to Keep Oral Health	● Tips to keep oral health ● Tooth brushing twice daily ● Daily flossing or interdental cleaning ● Healthy and balanced diet ● Regular dental check-ups ● Avoid smoking
10.	Smoking Cessation	● Smoking and periodontitis ● Tips for smoking cessation
11.	Alcohol Intake	● Alcohol intake & diabetes ● Tips for reducing alcohol intake
12.	Stress Management	● Tips for stress management ● Family support
13.	Fasting (religious and intermittent fasting)	● Tips for fasting for diabetic patients ● Conditions to break fast ● Dental care during fasting
14.	Hypoglycaemia	● Possible causes of hypoglycaemia ● Signs and symptoms of hypoglycaemia ● Hypoglycaemia management
15.	Hyperglycaemia	● Possible causes of hyperglycaemia ● Signs and symptoms of hyperglycaemia ● Hyperglycaemia management

**Table 2:** Socio-demographic characteristics of study participants

	Needs Assessment (*n* = 15)		Module Evaluation (*n* = 12)	
Characteristics	*n* (%)	Mean (SD)	*n* (%)	Mean (SD)
Age (years)		41.9 (8.0)		39.2 (6.9)
Gender				
Male	2 (13.3)		2 (16.7)	
Female	13 (86.7)		10 (83.3)	
Ethnicity				
Malay	13 (86.7)		11 (91.7)	
Chinese	2 (13.3)		1 (8.3)	
Profession				
Dietitian	5 (33.3)		4 (33.3)	
Dental Officer	2 (13.3)		1 (8.3)	
Dental Specialist	2 (13.3)		4 (33.3)	
Diabetic Educator	3 (20.0)		3 (25.0)	
Endocrinologist	1 (6.7)			
Pharmacist	1 (6.7)			
Health Psychologist	1 (6.7)			
Duration of Services (years)		15.0 (6.6)		13.5 (6.0)

Through the interview, most healthcare professionals agreed that an emphasis must be placed on the importance of optimal blood glucose maintenance through lifestyle modifications. This could be ensured via weight management, dietary intake, physical activity, hygiene care, smoking cessation, stress management, and compliance on medications and treatment plans. Particularly regarding dietary management, most of the participants highlighted the need for meal portion control to be included, especially carbohydrate intake. Sugar intake was constantly mentioned by dental and non-dental health professionals alike due to its substantial contribution to glycaemic control and oral health problems, such as dental caries and periodontitis. Common misconceptions about sugar, alcohol intake, and medication compliance were proposed to be included in the new module.

From the dental perspective, suggestions were made to emphasise dental plaque in the new module to create further awareness of the relationship between T2DM-periodontitis. Known as the primary etiology of bacterial infection-related oral diseases including periodontitis, both diseases involve inflammation and infection and may be caused by dental plaque. Oral hygiene care and self-examination upon noting the signs and symptoms of periodontitis among patients with diabetes were proposed for inclusion in the module.

Most participants suggested the new module be developed by incorporating fewer texts, the appropriate font sizes, and simpler layman terms to cater to readers’ literacy level within a diverse educational background. The use of pictures with captions and colourful illustrations would be preferable to ensure a clearer understanding of the new module and consider the different literacy levels among patients. Printed materials and appropriate as well as attractive audio-visual materials, preferably short videos, were proposed. Culturally relevant information was also suggested by participants as lifestyle advice is largely related to culture. Some participants recommended that common practice and local food should be included as part of the cultural component.

### Phase 2: module development

The developed module has 17 units consisting of 17 double-sided flip charts and 13 short videos (Table 3). Each unit of the flip chart has (i) a healthcare professional’s view, which consists of detailed explanations of the topic, and (ii) a patient’s view, which is more straightforward and mainly composed of pictures (infographics). The total number of pages in each flip chart unit is between 4 to 32 pages, including a front cover and an introduction section that describes the unit’s objective. Meanwhile, the average length of the video is 2.35 min and ended with a take-home message.

**Table 3:** Summary of module contents for the newly developed diabetes-periodontitis nutrition and health education module

Module	Module Unit (Flip chart)	No of Pages	Module Unit (Video)	Duration (min)
Module 1: Introduction to Diabetes and Periodontitis	Unit 1: Introduction to Type 2 Diabetes Mellitus (T2DM)	8	V1: Introduction to T2DM and Periodontitis	3.41
	Unit 2: Blood Sugar Monitoring	6	V2: Blood Sugar Monitoring	1.16
	Unit 3: Hypoglycaemia	7	V3: Hypoglycaemia and Hyperglycaemia	2.44
	Unit 4: Hyperglycaemia	4		
	Unit 5: Introduction to Periodontitis	6		
	Unit 6: Relationship between T2DM and Periodontitis	5		
Module 2: Diabetes and Periodontitis Care	Unit 7: Treatment for T2DM and Periodontitis	8		
	Unit 8: Oral Hygiene	9	V4: Oral Hygiene	2.58
	Unit 9: Additional Personal Effort on T2DM and Periodontitis Management	8	V5: Additional Personal Effort on T2DM and Periodontitis Management	3.31
Module 3: Lifestyle Modification	Unit 10: Weight Management	10		
	Unit 11: Healthy Eating	32	V6: Healthy Eating	2.32
			V7: Increase Dietary Fiber	3.03
			V8: Limit Sugar Intake	2.15
	Unit 12: Physical Activity and Exercise	10	V9: Physical Activity and Exercise	2.51
	Unit 13: Smoking Cessation	8	V10: Smoking Cessation	2.04
	Unit 14: Alcohol Intake	6	V11: Alcohol Intake	2.15
	Unit 15: Stress Management	6	V12: Stress Management	1.34
	Unit 16: Fasting	9		
Module 4: Myths and Facts	Unit 17: Myths and Facts of T2DM and Periodontitis	9	V13: Myths and Facts of T2DM and Periodontitis	2.07

### Phase 3: module evaluation

The expert panels comprised 12 healthcare professionals with a mean age of 39.2 (SD 6.9) years, ranging between 30 and 53 (Table 2). Most of the experts were female (83.3%) and of Malay ethnicity (91.7%).

#### Evaluation of the flip chart (PEMAT-P)

The overall median scores for understandability and actionability were 100%, ranging from 76.5 to 100% for understandability and 80 to 100% for actionability, respectively (Table 4). All experts scored 100% for Unit 9 and 16 for both understandability and actionability domain. Some feedbacks included straightforward and easy to understand and used attractive and appropriate visual aids. The expert panels also suggested reorganising a few medical and specific terms used in the flip charts and adding more detailed descriptions and explicit pictures.

**Table 4:** Scores of the understandability and actionability of printed materials (flip chart)

Module	Module Unit (Flip chart)	Understandability Score (%)		Actionability Score (%)	
		Median (IQR)	Min, Max	Median (IQR)	Min, Max
Module 1: Introduction to Diabetes and Periodontitis	Unit 1: Introduction to Type 2 Diabetes Mellitus (T2DM)	100.0 (0.0)	91.7, 100.0	100.0 (15.0)	80.0, 100.0
	Unit 2: Blood Sugar Monitoring	100.0 (0.0)	92.3, 100.0	100.0 (0.0)	–
	Unit 3: Hypoglycaemia	100.0 (3.8)	91.7, 100.0	100.0 (0.0)	80.0, 100.0
	Unit 4: Hyperglycaemia	100.0 (0.0)	81.8, 100,0	100.0 (0.0)	80.0, 100.0
	Unit 5: Introduction to Periodontitis	100.0 (0.0)	88.9, 100.0	100.0 (0.0)	–
	Unit 6: Relationship between T2DM and Periodontitis	100.0 (0.0)	90.9, 100.0	100.0 (0.0)	80.0, 100.0
Module 2: Diabetes and Periodontitis Care	Unit 7: Treatment for T2DM and Periodontitis	100.0 (0.0)	93.8, 100.0	100.0 (0.0)	80.0, 100.0
	Unit 8: Oral Hygiene	100.0 (0.0)	93.3, 100.0	100.0 (0.0)	–
	Unit 9: Additional Personal Effort on T2DM and Periodontitis Management	100.0 (0.0)	–	100.0 (0.0)	–
Module 3: Lifestyle Modification	Unit 10: Weight Management	100.0 (5.9)	93.8, 100.0	100.0, (0.0)	–
	Unit 11: Healthy Eating	100.0 (6.1)	76.5, 100.0	100.0 (0.0)	–
	Unit 12: Physical Activity and Exercise	100.0, 0.0	93.8, 100.0	100.0 (0.0)	–
	Unit 13: Smoking Cessation	100.0 (0.0)	91.7, 100.0	100.0 (0.0)	–
	Unit 14: Alcohol Intake	100.0 (0.0)	92.3, 100.0	100.0 (0.0)	–
	Unit 15: Stress Management	100.0 (0.0)	91.7, 100.0	100.0 (0.0)	80.0, 100.0
	Unit 16: Fasting	100.0 (0.0)	–	100.0 (0.0)	–
Module 4: Myths and Facts	Unit 17: Myths and Facts of T2DM and Periodontitis	100.0 (0.0)	–	100.0 (0.0)	80.0, 100.0

#### Evaluation of the short video (PEMAT-A/V)

The overall median scores for understandability and actionability were 100%, ranging from 90 to 100% for understandability (Table 5). All experts scored 100% for the actionability domain in all units. The videos were noted to be attractive, straightforward, and easy to understand. Further, the contents of the videos were consistent with the flip charts and compact with information. Adding voice-over to the videos and adding a demonstration video for oral hygiene topics were suggested to enhance the module’s understandability and actionability.

**Table 5:** Scores of the understandability and actionability of AV materials (video)

Module	Module Unit (Video)	Understandability Score (%)		Actionability Score (%)	
		Median (IQR)	Min, Max	Median (IQR)	Min, Max
Module 1: Introduction to Diabetes and Periodontitis	V1: Introduction to T2DM and Periodontitis	100.0 (0.0)	–	100.0 (0.0)	–
	V2: Blood Sugar Monitoring	100.0 (0.0)	90.0, 100.0	100.0 (0.0)	–
	V3: Hypoglycaemia and Hyperglycaemia	100.0 (0.0)	91.7, 100.0	100.0 (0.0)	–
Module 2: Diabetes and Periodontitis Care	V4: Unit 8: Oral Hygiene	100.0 (0.0)	–	100.0 (0.0)	–
	V5: Additional Personal Effort on T2DM and Periodontitis Management	100.0 (0.0)	–	100.0 (0.0)	–
Module 3: Lifestyle Modification	V6: Healthy Eating	100.0 (0.0)	–	100.0 (0.0)	–
	V7: Increase Dietary Fiber	100.0 (0.0)	–	100.0 (0.0)	–
	V8: Limit Added Sugar Intake	100.0 (0.0)	–	100.0 (0.0)	–
	V9: Physical Activity and Exercise	100.0 (0.0)	–	100.0 (0.0)	–
	V10: Smoking Cessation	100.0 (0.0)	–	100.0 (0.0)	–
	V11: Alcohol Intake	100.0 (0.0)	–	100.0 (0.0)	–
	V12: Stress Management	100.0 (0.0)	–	100.0 (0.0)	–
Module 4: Myths and Facts	V13: Myths and Facts of T2DM and Periodontitis	100.0 (0.0)	–	100.0 (0.0	–

## Discussion

Our present study successfully developed an integrated module for diabetes and periodontitis management that combined nutrition and health aspects. More importantly, the module was developed based on specific needs and interests expressed by the healthcare providers themselves. The module would serve as a structured diabetes education programme for healthcare professionals in Malaysia in providing care for diabetes and periodontitis. Besides, short educational videos can educate and empower patients with diabetes and oral health care needs and further improve their overall quality of life.

Studies have well established a bi-directional relationship between periodontitis-diabetes; diabetes is one of the risk factors for periodontitis and can exacerbate the severity of periodontitis, whereas periodontal inflammation will also have adverse effects on glycaemic control [27, 28]. Oral health education is important as it leads to improving oral health practices [29]. This module contains flip charts that healthcare professionals can use as an educational tool during counselling sessions with patients. Our previous needs assessment highlighted the lack of such educational materials or modules in the clinics (unpublished data). Besides, most non-dental healthcare professionals mentioned they missed the importance of oral health care and did not recognise periodontitis as a complication of diabetes. Hence, it is hoped that the developed flip charts can fill in the knowledge and practice gaps in this area amongst healthcare professionals.

It was found that most (95%) of patients with diabetes and periodontitis in Malaysia showed more interest in multimedia educational materials such as videos rather than printed materials [30]. In their study, the patients agreed that videos with a duration below 5 min are easier to comprehend and could spark their interest and retain focus and attention. Our study fulfilled this gap and produced videos with a limit of 3 min at most. Online video is easy to access and can convey the intended message to a broader audience range rather than a face-to-face method [31]. For health-related short video advertisements, informativeness, intrusiveness, relevance, and social interactions are the key factors for user acceptance [32]. High-quality online audio-visual educational material that is accurate, understandable, and actionable is warranted [33].

The current study used PEMAT to evaluate the educational module of both flip charts and videos. The PEMAT questionnaire is a systematic method that assesses two essential domains: understandability and actionability, with lower scores (70% and below) considered poorly understandable or poorly actionable [26]. Overall, the module’s median score for understandability and actionability was 100%, showing that most of the module’s unit is highly acceptable among healthcare professionals in the current study. The understandability scores demonstrated that the module was easy to understand and comprised clear and comprehensible contents. Layman terms were incorporated in the module instead of medical terms. If medical terms were used, subtitles and meaning were provided to avoid confusion. The module also included the use of appropriate visual aids. Meanwhile, the module’s actionability scores concluded that the module could clearly explain actions to be acted upon and directly referred to users.

Overall, our educational materials’ scores were higher than previously published health educational materials for diabetes (49.5% understandability and 31.4% actionability) [34] and oral health (71.5% understandability and 70% actionability) [35]. The higher scores of understandability and actionability reported in our study might be due to the participating healthcare professionals who are more familiar and expert in the areas. A slightly higher score for understandability (79.2%) and actionability (78.1%) was reported in a study among university students in evaluating printed material for smoking cessation [36].

The present study was the first study in Malaysia to integrate nutrition and oral health aspects in developing a diabetes-periodontitis module. This module was designed based on local and international established guidelines and considered healthcare professionals’ perspectives in managing T2DM and periodontitis. Our short videos, however, have yet to be evaluated by the patients. Of note, this study was conducted during the COVID-19 outbreak and Restriction Movement Order in the country. Therefore, the evaluation process was completed online with healthcare professionals, and the patients’ recruitment and access were limited.

## Conclusions

Our newly developed integrated nutrition and health education module for diabetes and periodontitis consisting of flip charts and short videos was highly well-received by expert panels. Future studies should evaluate this module’s use by healthcare professionals as one of the educational materials and its acceptance among diabetes and periodontitis patients.

## Data Availability

The datasets are available for interested researchers upon request from the corresponding author.

## Abbreviations

T2DM: Type 2 diabetes mellitus
COVID-19: Novel coronavirus disease
PEMAT-P: Patient education materials assessment tool for printable materials
PEMAT-A/V: Patient education materials assessment tool for audio-visual materials
